# Bacteriophage Lytic Enzyme P9ly as an Alternative Antibacterial Agent Against Antibiotic-Resistant *Shigella dysenteriae* and *Staphylococcus aureus*

**DOI:** 10.3389/fmicb.2022.821989

**Published:** 2022-02-14

**Authors:** Feng Wang, Yao Xiao, Yao Lu, Zheng-Yu Deng, Xian-Yu Deng, Lian-Bing Lin

**Affiliations:** ^1^Faculty of Life Science and Technology, Kunming University of Science and Technology, Kunming, China; ^2^Engineering Research Center for Replacement Technology of Feed Antibiotics of Yunnan College, Kunming, China

**Keywords:** bacteriophage, endolysin, bioengineering, antibiotic resistance, bacteriolytic activity

## Abstract

Developing new strategies to replace or supplement antibiotics to combat bacterial infection is a pressing task in the field of microbiological research. In this study, we report a lytic enzyme named P9ly deriving from the bacteriophage PSD9 that could infect multidrug-resistant *Shigella*. This enzyme was identified through whole-genome sequencing of PSD9. The results show that P9ly contains a conserved T4-like_lys domain and belongs to the phage lysozyme family. Recombinant P9ly obtained from protein purification presented biological activity and could digest bacterial cell walls (CW), resulting in the destruction of cell structure and leakage of intracellular components. Furthermore, P9ly exhibited bacteriolytic and bactericidal activity on different strains, especially multidrug-resistant Gram-negative *Shigella dysenteriae* and Gram-positive *Staphylococcus aureus*. Additionally, combined use of P9ly with ceftriaxone sodium (CRO) could decrease necessary dose of the antibiotic used and improve the antibacterial effect. In summary, under the current backdrop of extensive antibiotic usage and the continuous emergence of bacterial resistance, this study provides an insight into developing bacteriophage-based antibacterial agents against both Gram-negative and Gram-positive pathogens.

## Introduction

Bacteriophages are viruses that specifically infect bacteria and are mainly classified as virulent and temperate phage (Hobbs and Abedon, 2016). Virulent phages are specialized bacteria killers, while temperate phages can also follow the lytic pathway or propagate with the bacterial host as a prophage (lysogeny). Bacteriophages are not only the most abundant biological particles in the world, but also play an important role in the environment, as lysis of host cells can promote organic matter cycling and reduction in the counts of specific bacteria can regulate population equilibrium and biodiversity (Fernandez et al., 2021). In 2016, the Virus-X Consortium was established to explore genetic diversity and virions in extreme natural environments, further emphasizing essential roles of bacteriophages in bioprospecting and their importance as a source of new antimicrobial agents (Aevarsson et al., 2021).

In the 1920s to 1940s, bacteriophages were rapidly developed as effective antibacterial drugs and used to treat bacterial infections (Kutter et al., 2010). However, phage therapy was quickly abandoned in favor of antibiotics, which have broad antibacterial activity and are easy to prepare, store, and dispense. In recent years, the disadvantages of antibiotics have grown due to the emergence and spread of bacterial drug resistance (McCubbin et al., 2021). At present, multidrug-resistant bacteria are an extremely serious global problem. They are very difficult to treat, and a large number of antibacterial drugs have minimal inhibitory effects on these bacteria. Therefore, there is an urgent need to discover substitutes for traditional antibiotics.

Endolysins are usually a class of peptidoglycan (PG) hydrolases produced at the end of the replication cycle of bacteriophages and depend on host bacteria for synthesis (Fischetti, 2008). These lytic enzymes can rapidly cleave PG in bacterial cell wall (CW) and cause PG degradation, bulging of the cytoplasmic membrane and cell lysis, and subsequent release of progeny phages. As peptidoglycan is a major and essential component of the bacterial CW, there is a low likelihood of the host bacteria developing resistance to bacteriophage lytic enzymes in comparison with antibiotics (Allen et al., 2014).

Recently, bacteriophage lytic enzymes are widely reported as potential replacements to treat bacterial pathogens in different situations (Vázquez et al., 2018; Abdelkader et al., 2019). Studies have shown that the bacteriophage lytic enzyme LysEFm5 has a broader antibacterial spectrum than the bacteriophage encoding it, and it can lyse 19 out of 23 *Enterococcus* strains, including seven vancomycin-resistant strains (Gong et al., 2016). Bacteriophage lytic enzymes are also often used in controlling the foodborne bacteria. For example, the lytic enzyme LysH5 can effectively kill *Staphylococcus aureus* in milk (Obeso et al., 2008), while the lytic enzyme LysZ5 can effectively kill *Listeria monocytogenes* in soy milk (Zhang et al., 2012). Moreover, the extensive assays in animal models of infection or recent phase I/II clinical trials of lysin-based drugs further demonstrate the safety and efficacy of bacteriophage lytic enzymes in elimination of bacterial pathogens (Jun et al., 2014; Abdelkader et al., 2019).

It is well-known that the Gram-negative bacteria pose an outer membrane, and this difference in cell wall compared to Gram-positive species makes the former strains insensitive to destroy by exogenously added lysins (Allen et al., 2014). However, with the increasing discovery of lytic enzymes with intrinsic antibacterial activity against Gram-negative pathogens, this is no longer a no-go zone (Gutierrez and Briers, 2021). In this study, we investigated the lytic enzyme P9ly, encoded by the genome of bacteriophage PSD9 that infects multidrug-resistant *Shigella*. Gene cloning and the construction of an expression vector were used to obtain recombinant P9ly protein, and the antibacterial activity of P9ly toward different Gram-positive and Gram-negative strains was examined.

## Materials and Methods

### Bacterial Strains

*Shigella flexneri*, *Shigella sonnei*, *Salmonella* ser. Enteritidis, *Salmonella* ser. Paratyphi B, and *Escherichia coli*, which were used as targets for P9ly lysis, were purchased from the National Center for Medical Culture Collections (CMCC), and their strain numbers were CMCC(B)51572, CMCC(B)51592, CMCC(B)50335, CMCC(B)50094, and CMCC(B)44102, respectively. Multidrug-resistant *S. aureus* (1606BL1486) and *E. coli* O157 isolate (KUST401) were gifts from Professor Xueshan Xia and Professor Yuzhu Song, respectively, of the Molecular Medicine Research Center of Yunnan Province, Kunming University of Science and Technology. The antibiotic susceptibility of these strains was measured based on drug susceptibility tests following the guidelines provided by the US Clinical and Laboratory Standards Institute (CLSI). All other bacterial strains were from our lab collection. Among these strains, *E. coli* DH5a and BL21(DE3) were used for plasmid construction and expression of recombinant protein. *Escherichia coli*, *Staphylococcus*, *Shigella*, and *Salmonella* strains were cultured according to the previous published methods by our laboratory (Wang et al., 2020a) at 37°C with shaking (150 rpm). When required, 15 g/L agar or 7.5 g/L agar were added to the culture medium to obtain a solid or semi-solid culture medium. All of the aforementioned strains were stored in a −80°C freezer.

### Bacteriophage PSD9 Genome Sequencing and Annotation

Bacteriophage PSD9 was isolated and purified using the standard CsCl gradient centrifugation (Rodriguez-Rubio and Muniesa, 2021). Whole-genome sequencing of PSD9 was performed at Guangdong Magigene Biotechnology Co. Ltd., and the sequencing depth was more than 1,000×. MetaGeneMark was used for gene prediction and UniProtKB database for functional annotation (Zhu et al., 2010). Analysis of the conserved domains was based on the NCBI conserved domain database (CDD v3.19, last update: 8 March, 2021; Marchler-Bauer et al., 2017), and the results were further confirmed by using Pfam 35.0 (last update: 19 November 2021) and InterPro 87.0 (last update: 18 November 2021) databases (Blum et al., 2021; Mistry et al., 2021). The genome sequencing result has been uploaded to the NCBI GenBank database (accession number MZ561532.1).

### Production and Purification of Recombinant Protein P9ly

The following gene-specific primers were used for PCR amplification of the *P9ly* gene using the bacteriophage PSD9 genome as a template: forward primer: 5′-CATGCCATGGCAATGGATATTTTTGATATGTTACG-3′ and reverse primer: 5′-CGGAATTCTCTATAAGCAGCCCATGTGC-3′. The 5′ ends of the forward and reverse primers contained NcoI and EcoRI restriction sites (underlined in the primer sequences) that were used for amplification of the *P9ly* gene and directional cloning into the expression vector pET-28a. After the reaction system was thoroughly mixed, the amplification conditions were as follows: pre-denaturation at 94°C for 10 min; 30 cycles of denaturation at 94°C for 45 s, annealing at 58°C for 45 s, and extension of 72°C for 90 s; followed by a final extension at 72°C for 10 min. After the reaction was completed, 10 μl of PCR product was analyzed using 1% agarose gel electrophoresis, and the PCR product size was 486 bp. *Escherichia coli* BL21(DE3) containing the pET-28a-P9ly was used as the host cell for the expression of recombinant protein P9ly. Lactose (1 g/L) was used to induce the overexpression of the bacteriophage lytic enzyme P9ly. After lactose induction, the bacterial suspension was centrifuged at 12,000 × *g* for 10 min before the pellet was collected and then resuspended in the phosphate-buffered saline (PBS) containing 137 mM NaCl, 2.7 mM KCl, 4.3 mM Na_2_HPO_4_, and 1.4 mM KH_2_PO_4_ at pH 7.4. The device used for sonication was a Sonics VCX500 (Sonics & Materials Inc., United States). The sonication power setting was 120 W (0.5 s on and 0.5 s off). According to the manufacturer’s instructions, a HisTrap™ HP column (GE Healthcare, United States) was used for Ni-affinity purification of the recombinant protein P9ly, which was collected by eluting with imidazole-buffered saline (50–500 mM imidazole, 10 mM NaH_2_PO_4_, 10 mM Na_2_HPO_4_, and 500 mM NaCl). Subsequently, the pooled proteins were dialyzed against 20 mM Tris-HCl (PH 7.4) and 15% SDS-PAGE was used for analysis and validation of the P9ly protein.

### Bacterial Cell Wall Extraction

The cell walls were generated from *Shigella dysenteriae* KUST9, the host bacterium for phage PSD9, following the method published previously (de Jonge et al., 1992; Lim et al., 2014). In brief, the bacterial cells were cultured at 37°C until an OD_600_ of 0.6–0.8 was reached. Then, they were collected by centrifuging at 1,000 × *g* for 10 min at room temperature, washed twice with PBS buffer. Subsequently, the cells were transferred into 4% (final concentration) SDS and boiled for 30 min, and the cell walls were then concentrated by centrifugation for 5 min at 30,000 × *g*. The collected walls were further washed five times with PBS to remove remaining SDS. The preparation can be stored at −20°C for further analysis.

### Measurement of the Inhibitory Zones

This experiment was mainly performed according to the previously published paper (Li et al., 2021). In brief, 100 μl of logarithmic phase (OD_600_: 0.6–0.8) bacterial suspension and 4 ml LB semi-solid culture medium were mixed and plated on the prepared LB agar plate to form a double plate. An Oxford cup (diameter: 8 mm) was gently placed on a double plate using tweezers, and 200 μl of P9ly at a concertation of 112 μg/ml (6.05 μM) was added to the Oxford cup. The positive control was kanamycin (100 μg/ml) and the negative control was PBS buffer. The plates were cultured overnight at 37°C.

### Analysis of Antibacterial Activity of P9ly

In order to study the lytic activity of P9ly, different Gram-negative or Gram-positive bacterial strains were cultured and tested. In brief, these bacteria were cultured at 37°C and shaken at 150 rpm until the OD_600_ was 0.6–0.8. Following that, they were centrifuged at 4°C and 1,000 × *g* for 10 min. Subsequently, the bacteria were washed twice and then resuspended in PBS buffer. The reaction substrate and different concentrations (10, 30, and 50 μg/ml) of lytic enzyme P9ly were mixed evenly. As a note, the 0.54, 1.62 and 2.70 μM of P9ly are equivalent to the concentrations of 10, 30, and 50 μg/ml of the lysin, respectively. After that, a total volume of 200 μl was aspirated into a 96-well plate. The reaction mix between the substrate and PBS was used as the negative control. The plate was incubated in a thermostatic shaker at 37°C. The standard turbidity measurement method was used and the OD_600_ was recorded using a plate reader (Wisdom Applied Science, mode 6500, Newark, DE, United States) from 0 to 30 min (samples were collected every 5 min). Before measurement, all 96-well plates were shaken for 4 s for mixing. In the experiments, there was a parallel blank measurement that was subtracted from the values assayed. The bactericidal activity of P9ly was calculated as the relative reduction in logarithmic units after the indicated time points as follows: Log_10_ (N_0_/N_i_), N_0_ = number of untreated cells (in the negative control) and N_i_ = number of treated cells counted after P9ly incubation. The experiments were performed with at least three biological replicates.

### Combination of P9ly With Ceftriaxone Sodium

Three groups were set up for the experiment on combination between the lytic enzyme P9ly and ceftriaxone sodium (CRO). These included lytic enzyme P9ly alone (75 μg/ml, 4.05 μM), CRO alone (5 μg/ml, 7.56 μM), and 32.5 μg/ml lytic enzyme P9ly (2.025 μM) combined with 2.5 μg/ml CRO (3.78 μM). After incubation with different strains (count: 10^7^ CFU/ml) at 37°C for 60 min, 100 μl of the mixture was spread on an LB agar plate. The plates were inverted and incubated overnight in a 37°C thermostatic incubator. Then, changes in antibacterial activity among different groups were recorded and analyzed.

### Analysis of the Effects of pH, NaCl Concentration, and Temperature on P9ly Activity

The bacteriophage PSD9 host, *S. dysenteriae* KUST9 in the logarithmic growth phase, was used as the substrate for P9ly lysis. The measured pH range was 5–10, the NaCl concentration was 0–600 mM, and the temperature range was 4–60°C. The lytic activity of P9ly under specific conditions was calculated as [OD_600_ (buffer only)—OD_600_ sample (lysin added)]/initial OD_600_, as described previously (Plotka et al., 2015, 2019a). Three biological replicates were used for every measurement.

### Scanning Electron Microscopy

For scanning electron microscopy, 10^5^ CFU/ml suspensions of different bacterial strains were centrifuged at 1,000 × *g* for 5 min to collect the bacteria. The bacteria were washed thrice with PBS buffer. In the experimental group, 1 ml P9ly solution (75 μg/ml, 4.05 μM) was added. In the control group, 1 ml PBS was added. The solutions were incubated in a 37°C thermostatic incubator for 1 h. After that, the solutions were centrifuged, the supernatants were discarded, and the pellets were collected. In every tube, 2 ml 2.5% glutaraldehyde was added and fixation was carried out at 4°C for 6 h. The tubes were centrifuged for 5 min to collect the pellet and the supernatant was discarded. The pellet was washed thrice with PBS. Then, 30, 50, 60, 70, 80, and 90% ethanol were prepared, and dehydration was performed with the lowest to highest concentration of ethanol, once each, followed by two rounds of dehydration using absolute ethanol. During dehydration, 1 ml of each ethanol concentration was added at a time and incubated at room temperature for 20 min, followed by centrifugation for 5 min, after which the supernatant was discarded. Bacteria were evenly applied to a 10 × 10 mm silicon slide and air-dried for 24 h. After that, scanning electron microscopy was performed according to the manufacturer’s instructions (model no. FlexSEM1000, *Hitachi* High Tech Group, Japan).

### Statistical Analysis

The R software was used and the Student’s *t*-test was employed to determine statistical differences between quantitative data. All data were expressed as mean ± SD (*n* = 3 independent experiments). A difference of two-tailed *p* < 0.05 was considered to be statistically significant.

### Nucleotide and Protein Sequence Accession Numbers

The whole-genome sequence of bacteriophage PSD9 was deposited in the NCBI GenBank database with the accession number MZ561532.1, and the protein sequence of bacteriophage lytic enzyme P9ly was stored under the accession number QXV72475.1.

## Results

### Identification of P9ly From the Bacteriophage PSD9 Genome

**Figure 1:**
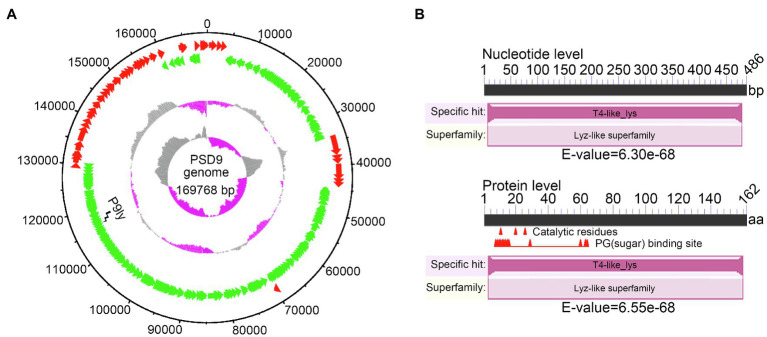
Whole-genome sequencing of bacteriophage PSD9 was carried out to identify the lytic enzyme P9ly encoded by it. **(A)** Bacteriophage PSD9 genome map. The inner to outer circles are as: GC skew [(G − C)/(G + C)]; GC content percentage; negative strand open reading frames (ORFs; shown in green); positive strand ORFs (shown in red); and genome size (with an interval of 10 kb). **(B)** Conserved domain analysis of P9ly at nucleic acid and protein levels.

Previously, we have isolated a phage infecting multidrug-resistant *S. dysenteriae* strain KUST9 (GenBank accession: MZ452143.1) in chicken intestines from a chicken farm in Guangxi province, China, and named it the phage PSD9, which is a lytic virus belonging to the family of Myoviridae. In this study, we further carried out whole-genome sequencing on the phage (GenBank accession: MZ561532.1). Figure 1A shows that the PSD9 genome is 169,768 bp and its GC content is 39.46%. Bioinformatics analysis has shown that the PSD9 genome encodes 261 proteins (of which 199 proteins have known functions). The corresponding lytic enzyme gene of this genome is ORF190 (base length: 486 bp). In order to facilitate following description, we named this lysin as P9ly (GenBank accession: QXV72475.1). P9ly consists of 162 amino acids and its similarity to the ENLYS_BPT4 (Swiss-Prot ID: P00720) sequence is 74.1% (E-value: 6.55e−87). This protein contains a conserved T4-like_lys domain (NCBI domain architecture ID: 10091399; conserved domain accession: cd00735) and belongs to the lyz-like superfamily (Figure 1B). Consistently, the functional domain present in this lysin was also predicted to belong to the phage lysozyme family (PF00959, E-value: 9.20e−21) and glycoside hydrolase, family 24 (IPR002196, E-value: 8.60e−21) according to Pfam (Mistry et al., 2021) and InterPro (Blum et al., 2021) search results, respectively. Based on these findings, we conclude that P9ly could be a T4-like lysozyme with N-acetylmuramidase activity that cleaves the β(1,4) glycosidic bond between the C1 of N-acetylmuramic acid (MurNAc) and C-4 of N-acetylglucosamine (GlcNAc) in the bacterial peptidoglycan heteropolymers (Loessner, 2005).

**Figure 2:**
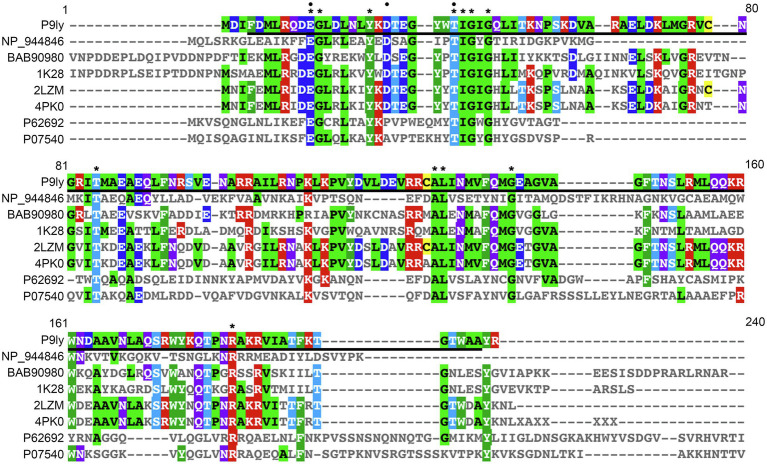
Multiple sequence alignment of lytic enzyme P9ly and other seven representative members of the lyz-like family. This analysis was performed using the EBI tool CLUSTAL Omega 1.2.4. In the figure, a star denotes conserved amino acid residues among all the sequences, while a black circle indicates critical residues that built the enzyme’s catalytic center. Bold underlined regions show the position of the T4-like_lys domain in the lytic enzyme P9ly. The accession numbers of NP_944846, BAB90980, 1K28, 2LZM, 4PK0, P62692, and P07540 represent protein sequences of the lysozyme from *Salmonella* phage FelixO1 (GenBank ID: 38707815), tail lysozyme from *Escherichia* phage RB49 (GenBank ID: 20218971), chain A, tail-associated lysozyme from *Escherichia* virus T4 (GenBank ID: 18655470), chain A, T4 lysozyme from *Escherichia* virus T4 (GenBank ID: 157835331), chain A, lysozyme from *Actinoplanes teichomyceticus* (GenBank ID: 683437436), endolysin from *Lactococcus* virus c2 (GenBank ID: 50402201), and endolysin from *Bacillus* phage PZA (GenBank ID: 126604), respectively.

Further, we performed multiple sequence alignment between P9ly and seven representative members of the lyz-like family by employing Clustal Omega (version 1.2.4; Sievers and Higgins, 2018). These representative members were chosen based on sequence cluster and sub-family hierarchy among endolysins with the conserved domain of T4-like_lys (domain id: cd00735, type selection: top listed sequences). As shown in Figure 2, we found that P9ly protein contained 12 completely conserved residues (E11, G12, 18Y, T26, I27, G28, G30, T59, A98, L99, G107, and R145). Interestingly, we also found a highly conserved TIGIG motif at the N-terminal of the conserved T4-like_lys domain of P9ly. We speculate that this motif may participate in peptidoglycan binding and catalyze the degradation process.

### Molecular Cloning, Expression, Purification, and Activity Validation of Recombinant Lytic Enzyme P9ly

**Figure 3:**
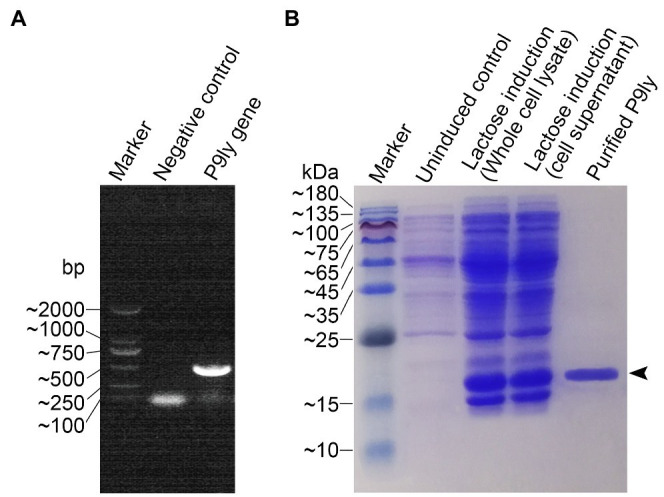
Gene cloning, recombinant protein expression, and purification of the lytic enzyme P9ly. **(A)** The bacteriophage PSD9 genome was used as a template for PCR amplification of the *P9ly* gene. **(B)** A final concentration of 1 g/L lactose was used to induce overexpression of recombinant P9ly, and the final purified recombinant lytic enzyme P9ly was analyzed using 15% SDS-PAGE. Black arrows indicate the protein band of the recombinant P9ly.

**Figure 4:**
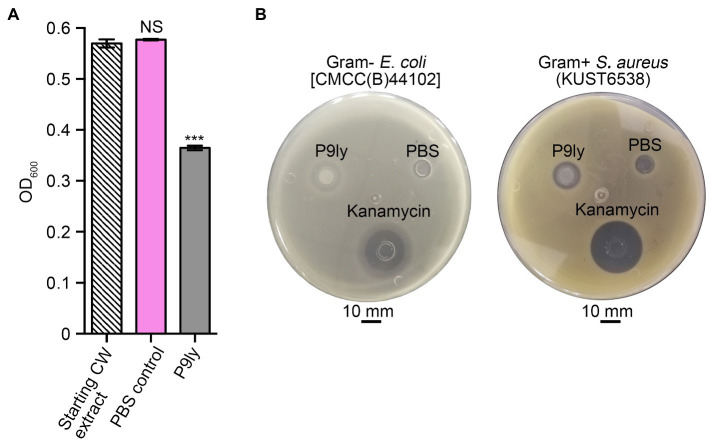
Validation of the biological activity of recombinant P9ly, which manifests as bacterial cell wall (CW) digestion and the exertion of inhibitory effects toward Gram-negative *Escherichia coli* and Gram-positive *Staphylococcus aureus*. **(A)** P9ly can digest the CW obtained from *Shigella dysenteriae* KUST9, the host bacterium for phage PSD9. **(B)** Inhibitory zones of recombinant P9ly against Gram-negative *E. coli* and Gram-positive *S. aureus*. Phosphate-buffered saline (PBS) buffer and 100 μg/ml kanamycin were used as negative and positive controls, respectively. *** indicates *p* < 0.001; NS, not significant.

As shown in Figure 3A, we used PCR amplification to obtain the P9ly gene sequence from the genome of bacteriophage PSD9. Lactose-induced expression of recombinant lytic enzyme P9ly was carried out in genetically engineered *E.coli* BL21 (DE3). The HisTrap affinity column was used for protein affinity purification (Figure 3B), and we further employed 15% SDS-PAGE to confirm the band position and purity of purified recombinant P9ly. Following that, we validated the bacterial cell wall lytic activity of recombinant lytic enzyme P9ly. As shown in Figure 4A, the cell walls of the bacteriophage PSD9 host, *S. dysenteriae* strain KUST9, were used as the substrate to demonstrate that P9ly could digest it. The inhibitory zone experiment further illustrated that the recombinant lytic enzyme P9ly showed inhibitory activity against both Gram-negative *E. coli* and Gram-positive *S. aureus*, with corresponding inhibitory zone diameters of 13 and 12 mm, respectively (Figure 4B). Compared with the negative control, the inhibitory zones of the *E. coli* and *S. aureus* strains increased by 62.5 and 50%, respectively, after treatment with the lytic enzyme P9ly.

### Measurement of the Effects of Different Experimental Conditions on Lytic Enzyme P9ly Activity

**Figure 5:**
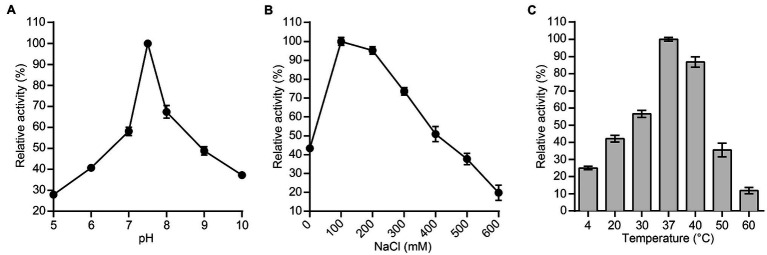
Effects of different experimental conditions on lytic enzyme P9ly activity. **(A)** pH; **(B)** NaCl concentration; and **(C)** Temperature. *Shigella dysenteriae* KUST9, the host bacterium for bacteriophage PSD9, was used as an effector substrate for lytic enzyme P9ly. In addition, the maximum activity of P9ly under various test conditions was set as 100% to facilitate comparison. Every experiment was repeated in triplicate. The error bars represent the SD.

Furthermore, we used the host bacterium of bacteriophage PSD9, *S. dysenteriae* strain KUST9, as the effector substrate for the lytic enzyme P9ly to measure the effects of different pH values, NaCl concentrations, and temperatures on its activity. The pH values tested ranged from 5 to 10, and P9ly had the highest activity at pH 7.5 (Figure 5A). Based on the result, we used this most suitable pH to test the effects of NaCl concentration on P9ly activity. As shown in Figure 5B, the optimal NaCl concentration for P9ly was found to be 100 mM. As the NaCl concentration under normal physiological conditions in the human body is around 130 mM, this result provides a basis for the *in vivo* application of P9ly. In addition, we measured P9ly lytic activity within the 4–60°C temperature range. Figure 5C shows that P9ly has the highest activity at 37°C, which is the optimal growth temperature for many bacterial strains.

### Analysis of Bacteriolytic and Bactericidal Activity of P9ly on Different Pathogenic Bacteria

**Figure 6:**
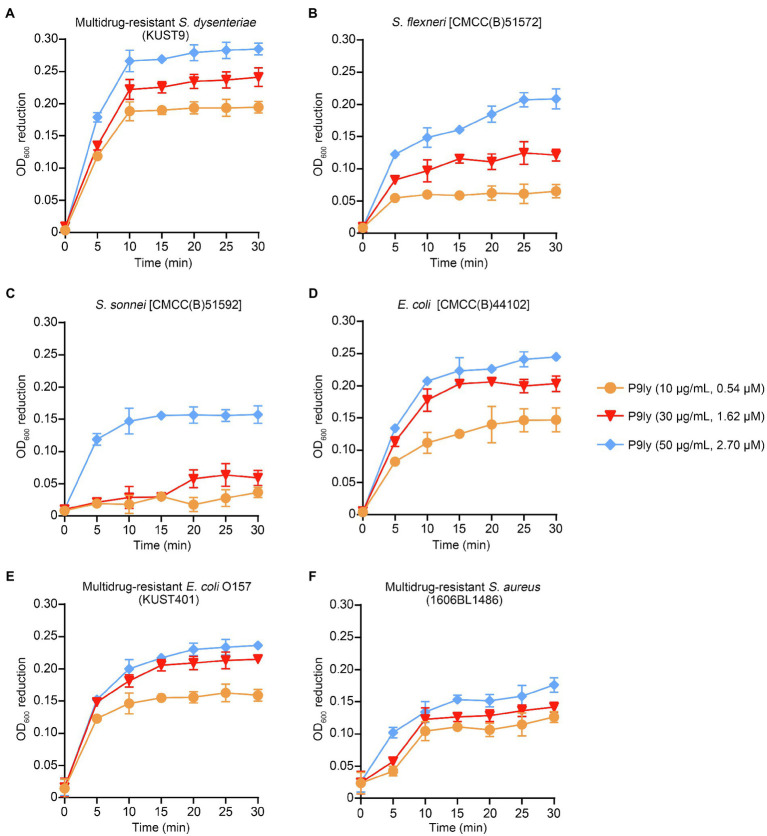
Dependence of P9ly bacteriolytic activity on its concentration. Standard turbidity reduction assays were used to measure the effects of lytic enzyme P9ly under different treatment durations and different concentrations. The various panels in the figure show that P9ly acts on different strains. **(A)** Multidrug-resistant *Shigella dysenteriae* (KUST9); **(B)** *Shigella flexneri* [CMCC(B)51572]; **(C)** *Shigella sonnei* [CMCC(B)51592]; **(D)** *Escherichia coli* [CMCC(B)44102]; **(E)** Multidrug-resistant *E. coli* O157 (KUST401); and **(F)** Multidrug-resistant *Staphylococcus aureus* (1606BL1486). It should be noted that there was a blank measurement subtracted from the assayed values in the experiment. For all strains, OD600 reduction was calculated in comparison with PBS-treated control at each time point. Values are expressed as mean ± SD. The experiment was repeated in triplicate.

As shown in Figure 6, we studied the dose-dependent lytic activity of P9ly on six different pathogenic bacterial strains (multidrug-resistant *S. dysenteriae* strain KUST9, *S. flexneri*, *S. sonnei*, *E. coli*, multidrug-resistant *E. coli* O157, and multidrug-resistant *S. aureus*) under 37°C. We found that the bacteriolytic activity of P9ly increased proportionally as the concentration increased and exhibited dose dependence. In addition, the P9ly bacteriolysis curve showed rapid kinetics, and it is worth noting that at a P9ly concentration of 50 μg/ml (2.70 μM), maximum turbidity decrease is generally reached very quickly (after 5–10 min incubation). The *p*-values of OD_600_ reduction upon 50 μg/ml of P9ly treatment for 5 min in comparison with PBS-treated controls were 3.04e−06, 1.18e−05, 2.14e−04, 6.88e−07, 9.28e−06, and 5.81e−04 for multidrug-resistant *S. dysenteriae*, *S. flexneri*, *S. sonnei*, *E. coli*, multidrug-resistant *E. coli* O157, and multidrug-resistant *S. aureus*, respectively. For multidrug-resistant *S. dysenteriae*, an OD_600_ reduction of 0.27 could be achieved upon a 10 min incubation of P9ly with a concentration of 50 μg/ml (2.70 μM), and the *p*-value was 7.18e−05 compared with PBS-treated control.

**Figure 7:**
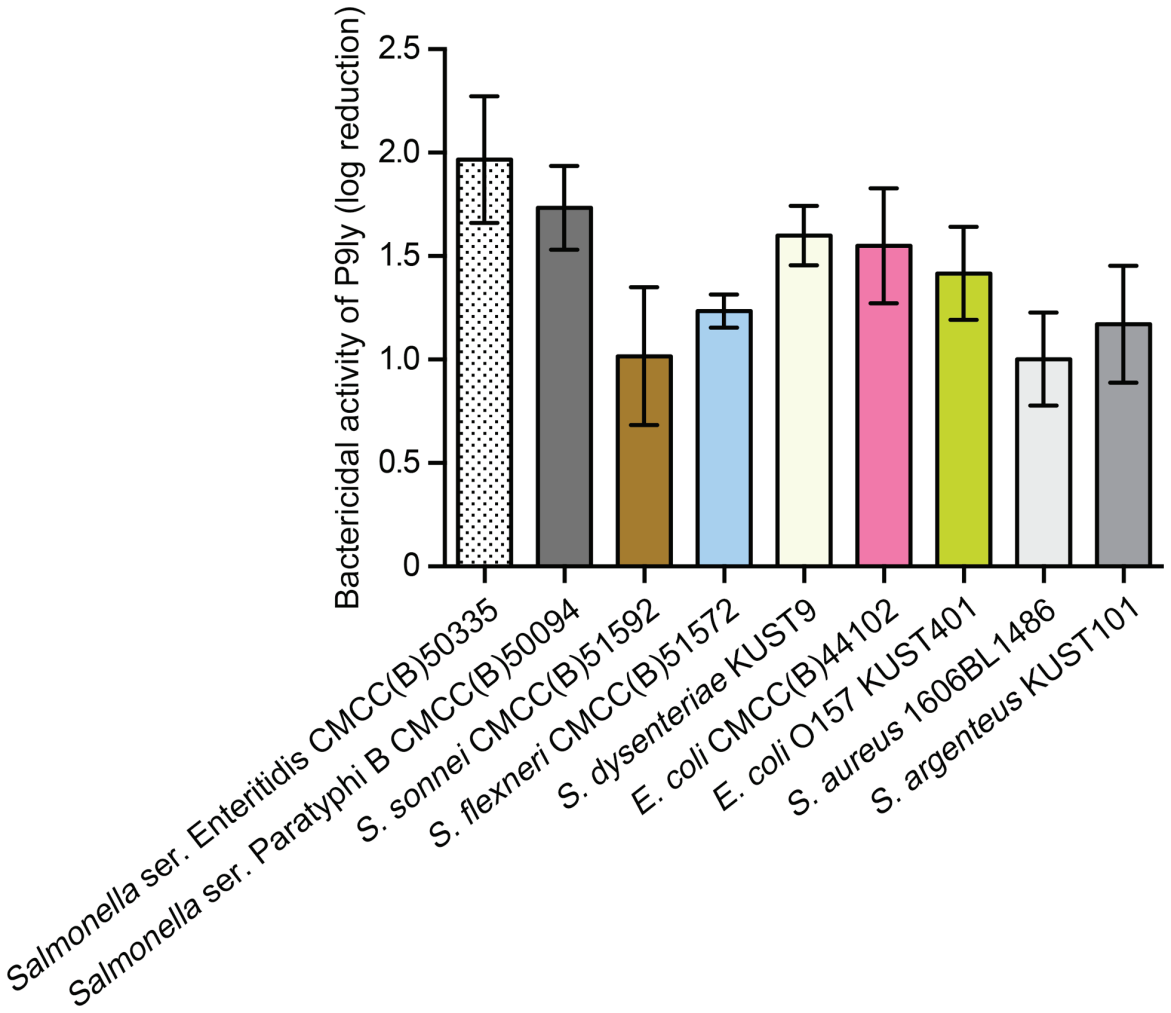
Bactericidal activity of P9ly against different bacterial strains. Among them, *Staphylococcus aureus* (1606BL1486) and *Staphylococcus argenteus* (KUST101) are Gram-positive bacteria. Approximately 10^7^ CFU/ml of different strains were treated with 75 μg/ml (4.05 μM) of P9ly at 37°C for 60 min, log kills were evaluated as the relative decrease in the viable counts of bacterial cell suspensions. All data were expressed as mean ± SD of three biological replicates.

Moreover, we measured bactericidal activity of P9ly against different pathogenic strains as a relative decrease in logarithmic units. It can be seen from Figure 7 that P9ly at a concentration of 75 μg/ml (4.05 μM) showed killing activity against all these strains including Gram-negative and Gram-positive ones, with decreasing values in viable cell counts ranging from approximate 1 to 2 log reduction. In summary, these results showed that P9ly could kill both Gram-negative and Gram-positive bacteria, demonstrating its potential antibacterial application value in biotechnology.

### Scanning Electron Microscopy of Lysis in Different Pathogenic Bacteria by P9ly

**Figure 8:**
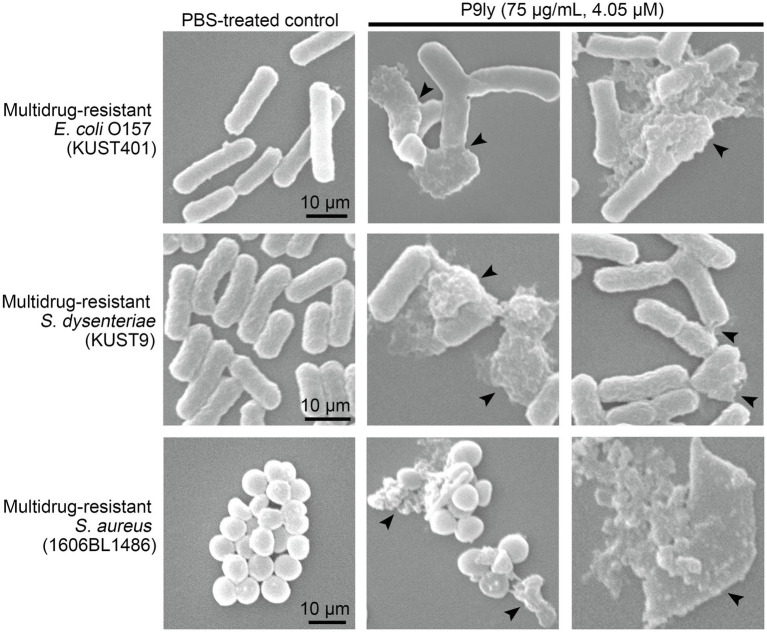
Scanning electron microscopy was used to observe lysis in multidrug-resistant Gram-negative bacteria (*Escherichia coli* O157 and *Shigella dysenteriae* KUST9) and Gram-positive bacteria (*Staphylococcus aureus* 1606BL1486) after P9ly treatment. In all experiments, approximately 10^5^ CFU/ml of different bacterial strains were incubated with 75 μg/ml (4.05 μM) P9ly or PBS control at 37°C for 60 min. In the figure, black arrows indicate the bacteriolytic effects of P9ly on bacterial cells. The scale bar for each bacterial strain is 10 μm.

To further study the morphological changes in bacterial cells after P9ly treatment, we employed scanning electron microscopy to observe lysis in multidrug-resistant Gram-negative bacteria (*E. coli* O157 and *S. dysenteriae* KUST9) and Gram-positive bacteria (*S. aureus* 1606BL1486) after P9ly treatment. As shown in Figure 8, scanning electron microscopy photographs showed that compared with PBS control, significant destruction and leakage of intracellular contents occurred in these bacterial strains after treatment with 75 μg/ml (4.05 μM) P9ly at 37°C for 60 min, suggesting that exogenous P9ly, a lytic enzyme from bacteriophage PSD9, can disrupt the cell walls of these bacteria and cause leakage of intracellular contents, thereby killing the bacteria. These observations were consistent with the above antibacterial activity results of P9ly, further demonstrating that recombinant P9ly is valuable as a functional bacteriophage lytic enzyme.

## Discussion

In recent years, the continuous and rapid emergence of multidrug-resistant bacteria has caused bacteriophages and their lytic enzymes to gradually attract attention as antibiotic substitutes for treating pathogenic bacterial infections (Donovan, 2007; Maciejewska et al., 2018). Although bacteriophages have returned to medical treatment, the host spectrum of them is usually narrow, and therefore, multi-phage cocktails are frequently required in treatment (Maciejewska et al., 2018). For lytic enzymes encoded by bacteriophages, their specificity and host range vary. Due to strong variation in peptidoglycan structure and glycan chain among Gram-positive bacteria, the activity of lysins from Gram-positive-specific phages can be limited to certain genus, species, or even serotype (Briers and Lavigne, 2015). However, under some circumstances, this narrowness allows for selective elimination of a pathogenic target, keeping associated microflora unaffected and lowering development of bacterial resistance. On the contrary, great similarity of peptidoglycan structure shares among Gram-negatives, and thus, a lytic enzyme derived from a phage infecting Gram-negative bacteria is usually active against a broad host range (Maciejewska et al., 2018).

In this study, bacteriophage PSD9, which was isolated from chicken intestines in a chicken farm, was used as a material and its genome was sequenced and analyzed. In line with the virulent feature of PSD9, we did not find lysogeny module in its genome (GenBank numbers: MZ561532.1). In many cases, the lysis module of the bacteriophages is composed of several genes grouped in a single operon, and the endolysin gene often follows a holin gene in the genome (Pasqua et al., 2021). Indeed, a holin gene (ORF57) could be detected in the genome of PSD9. Unexpectedly, the annotated endolysin gene of PSD9 was identified as ORF190 (namely, the P9ly gene), which is far from the holin gene. Subsequently, the P9ly encoded by the bacteriophage PSD9 genome was cloned and expressed and underwent induction and purification to obtain the recombinant protein. The antibacterial and antibiotic dose-reducing activity of this recombinant lysin was analyzed and confirmed. Of note, recombinant expression of P9ly in *E. coli* without coproduction of a holin indeed led to cell lysis, suggesting that P9ly has the ability to pass the cytoplasmic membrane independently, which is very similar to the endolysin Lys1521 of a *Bacillus amyloliquefaciens* phage (Morita et al., 2001). Overall, these results are consistent with organization of the lysis module in PSD9 genome and deserve further investigation.

Due to the presence of an outer membrane in Gram-negative bacteria, many antibiotics cannot penetrate the cells to exert their effects (Briers and Lavigne, 2015). This causes many studies to focus on the screening and identification of antibacterial agents that can target and kill Gram-negative bacteria to substitute or complement traditional antibiotics (Lim et al., 2014). However, the number of bacteriophage lytic enzymes reported to kill Gram-negative bacteria is far lower than that reported to kill Gram-positive bacteria (Trudil, 2015; Maciejewska et al., 2018). In this study, we found that the lytic enzyme P9ly could kill both Gram-negative bacteria (such as *Shigella* and *E. coli*) and Gram-positive bacteria (such as *S. aureus*). Given that P9ly belongs to a family of lysozymes, we speculate a reason for its broad activity could be that the target is indeed the glycan chain of the peptidoglycan, which is the most conserved part of it.

Recently, Plotka et al. (2019b) have highlighted that a promising antimicrobial endolysin Ts2631 (GenBank: AIM47292, 156 aa) derived from the *Thermus* phage vB_Tsc2631 has a unique N-terminal extension (residues 1–20), and this highly positively charged 20-residue N-terminus is of great importance for the enzyme to pass the outer membrane and then function in substrate anchoring (Plotka et al., 2019b). We compared the protein sequence between Ts2631 and P9ly, especially their N-terminal part, but no significant similarity was found. Additionally, several studies (Thandar et al., 2016; Sykilinda et al., 2018; Vázquez et al., 2021a,b) have revealed that antimicrobial peptide (AMP)-like regions in the C-terminal of phage lysins and/or their alpha-helical structure exposed outside of the protein globule showed superb antimicrobial activity and could be engineered for improved bactericidal activity. However, we also did not find similarity between P9ly and these reported C-terminal AMPs, including PlyF307 (Thandar et al., 2016), AcLys (Sykilinda et al., 2018), and Pae87 (Vázquez et al., 2021a). Following comparison between P9ly and molecule structures of the bacteriophage T4 lysozymes (Kanamaru et al., 2002; Arisaka et al., 2003; Han et al., 2014) based on NCBI conserved domain database, we found three critical residues that built the enzyme’s catalytic center, namely, E11, D20, and T26 (Figures 1B, 2), while the key peptidoglycan binding sites in the amino acid sequence of P9ly were identified as R8, Q9, D10, E11, G12, L13, D14, L15, N16, I29, M60, A63, and E64. Thus, we hypothesize that the continuous nine-residue of RQDEGLDLN at the N-terminus of P9ly is greatly important for its peptidoglycan binding activity. For the highly conserved TIGIG motif at the N-terminal of P9ly, the critical T26 and I29 residues coexist and are predicted to be responsible for catalytic and peptidoglycan binding activities, respectively. Site-directed mutagenesis needs to be performed to fully uncover the biological function of this TIGIG motif in P9ly. Overall, it seems that the 64-residues at the N-terminus of P9ly are both necessary for cell lysis and crucial for peptidoglycan binding, which is consistent with the fact that 93.9% of the endolysins derived from phages infecting Gram-negative bacteria are globular (Briers and Lavigne, 2015). Actually, this type of architecture carrying a single phage lysozyme domain also accounted for 50.8% of the discovered lysins from phages infecting Gram-negative bacteria in a recently constructed database (Vázquez et al., 2021b). In the future, protein structure-related studies could be performed to elucidate the molecular mechanism of bacteriophage lytic enzyme P9ly more accurately from a structural perspective.

Interestingly, we found through SMS2 software (Stothard, 2000) prediction that the isoelectric point (pI) of P9ly was 10.23. As we know, the bacterial surface is usually negatively charged due to high content of lipid and existence of secondary cell wall polymers (SCWPs), which are associated with the lipid bilayer and PG layer (Low et al., 2011). Lines of literatures (Foreman-Wykert et al., 1999; Buckland et al., 2000; Donovan et al., 2006a,b) have presented that the correlation between positive net charge on lysin catalytic domains and their bactericidal activity as well as lytic spectrum, demonstrating that the key role of positive charge on phage lytic enzymes in penetrating the bacterial PG layer, which is especially true for a lytic enzyme with small single domain and high positive net charge. Given the aforementioned domain characteristics and high pI of P9ly, we propose that the electrostatic interaction between P9ly and bacterial surface could facilitate localization of the enzyme, possibly through electrostatic steering (Kozack et al., 1995), thereby enabling it to penetrate the barrier of the cell wall, and hence be responsible for observed P9ly antibacterial activity in this study. Although net charge is not the only force in determining the way of lysins to the cell wall, these findings do indicate a facile approach for fine-tuning lytic activity of P9ly.

Preliminarily, we also studied the antibacterial activity of P9ly combined with CRO, which is a cephalosporin antibiotic with broad spectrum and is used in the treatment of pneumonia, bronchitis, peritonitis, infections of the skin, and meningitis caused by susceptible bacteria (Gregoire et al., 2021). As shown in Supplementary Figure S1, compared with P9ly (75 μg/ml, 4.05 μM) or CRO (5 μg/ml, 7.56 μM) individual treatment, we observed improved log kills when the concentrations of both agents were halved and used together (32.5 μg/ml P9ly + 2.5 μg/ml CRO), suggesting that combination between P9ly and CRO could considerably decrease the necessary dose of the antibiotic used. We believe that this halved combination is valuable if its antibacterial effect is not reduced with respect to antibiotic monotreatment (maybe it is still acceptable even if the combination has equal or similar antibacterial effect), and combined use of the two antibacterial agents with very distinct modes of action (an endolysin and an antibiotic) might decrease the chances of antibiotic resistance development. However, additional data are still needed to uncover the best proportion between P9ly and CRO, and to distinguish between a synergistic or additive effect resulting from the combined action of the two agents, for example, by determination of their fractional inhibitory concentrations (FICs) and calculation of the FIC indices (Huang et al., 2019).

In addition, we frequently observed that the bactericidal effect of the lytic enzyme slowly decreased with increasing storage duration in the freezer. Indeed, low stability and an inactivation tendency are major shortcomings of protein antibacterial agents (Wang et al., 2020b). These shortcomings should be the focus of future research, and efforts should also be made to improve the stability and storage life of these agents.

## Conclusion

We report here the identification, production, and characterization of the lytic enzyme P9ly, which derived from the natural bacteriophage PSD9, fighting against the antibiotic-resistant bacterial pathogens including multidrug-resistant Gram-negative *Shigella dysenteriae* (to our knowledge, *Shigella* pathogens remain rather unexplored as a target for lysins) and Gram-positive *S. aureus*. Moreover, halved combination between P9ly and CRO presented improved antibacterial effect. In the current age of mounting antibiotic resistance, P9ly exhibits great potential for replacing or supplementing traditional antibiotics.

## Data Availability Statement

The datasets presented in this study can be found in online repositories. The names of the repository/repositories and accession number(s) can be found in the article/Supplementary Material.

## Author Contributions

L-BL, X-YD, and FW conceived and designed the study. YX, YL, and Z-YD performed the experiments. FW and YX analyzed the data. FW performed the statistical analysis and drafted the manuscript. L-BL and FW revised the manuscript. All authors contributed to manuscript revision, read and approved the submitted version.

## Funding

This work was supported by the Yunnan Fundamental Research Projects (grant nos. 202001AT070048 and 202101AT070122).

## Conflict of Interest

The authors declare that the research was conducted in the absence of any commercial or financial relationships that could be construed as a potential conflict of interest.

## Publisher’s Note

All claims expressed in this article are solely those of the authors and do not necessarily represent those of their affiliated organizations, or those of the publisher, the editors and the reviewers. Any product that may be evaluated in this article, or claim that may be made by its manufacturer, is not guaranteed or endorsed by the publisher.

## Supplementary Material

Supplementary Figure S1 Combination of P9ly with ceftriaxone sodium (CRO) against different bacterial pathogens. **(A)** *Shigella dysenteriae* (KUST9); **(B)** *Shigella flexneri* [CMCC(B)51572]; **(C)** *Staphylococcus aureus* (KUST6538); and **(D)** *Staphylococcus argenteus* (KUST101). The doses of P9ly and CRO were halved before they were used in combinational treatment. Data are expressed as mean ± SD. *p* values were calculated based on Student’s *t*-test, ^*^*p* < 0.05, ^**^*p* < 0.01.
