# Supplementary figures and images for: Bacteriophage Lytic Enzyme P9ly as an Alternative Antibacterial Agent Against Antibiotic-Resistant Shigella dysenteriae and Staphylococcus aureus
Source: Front Microbiol. 2022 Feb 14;13:821989. doi: 10.3389/fmicb.2022.821989 (PMC8882861; doi:10.3389/fmicb.2022.821989)

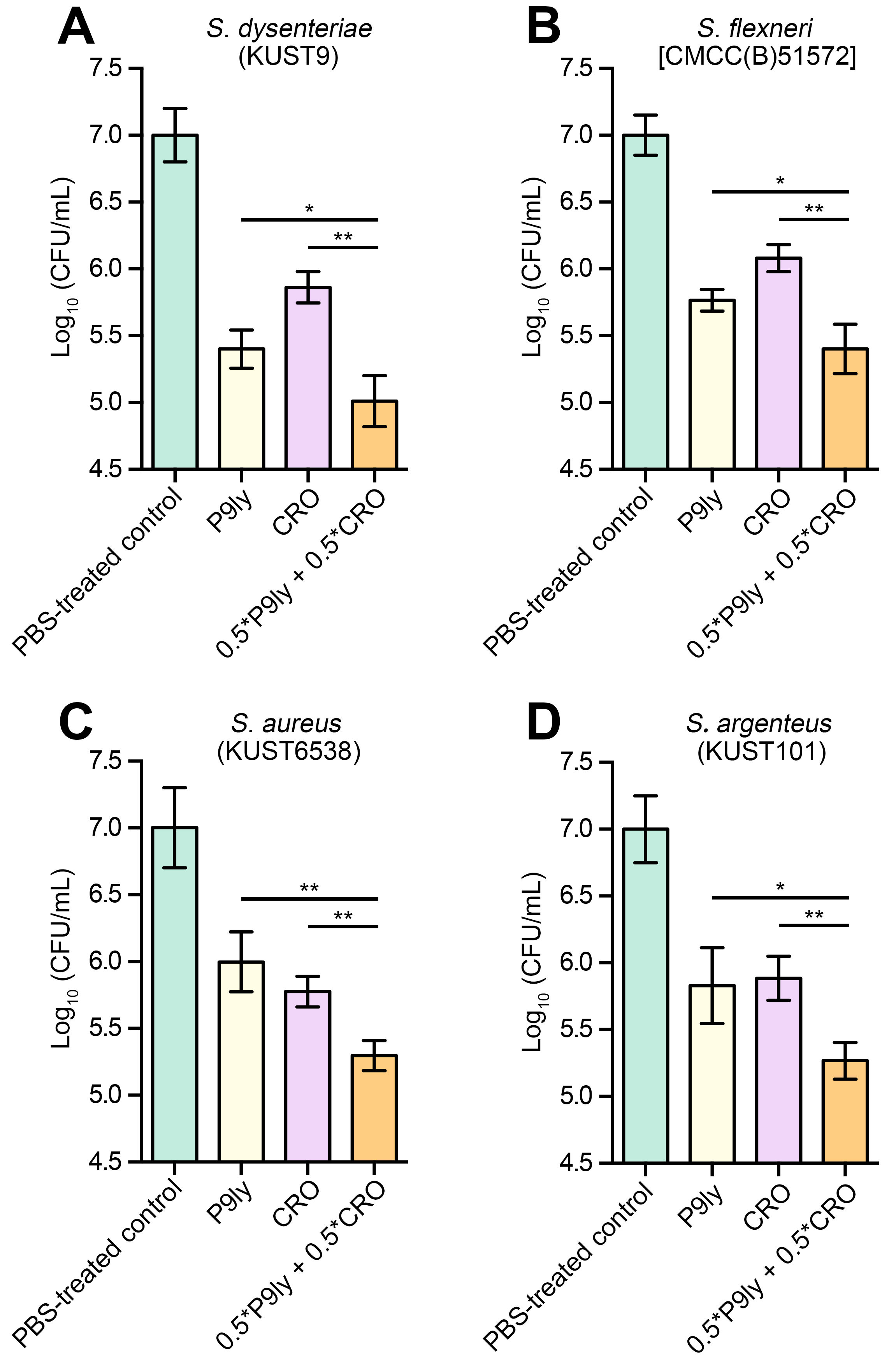

Supplement: Supplementary Figure S1 — Combination of P9ly with ceftriaxone sodium (CRO) against different bacterial pathogens. (A) Shigella dysenteriae (KUST9); (B) Shigella flexneri [CMCC(B)51572]; (C) Staphylococcus aureus (KUST6538); and (D) Staphylococcus argenteus (KUST101). The doses of P9ly and CRO were halved before they were used in combinational treatment. Data are expressed as mean ± SD. p values were calculated based on Student’s t-test, *p < 0.05, **p < 0.01. [file Image_1.TIF]
